# The first reported case of intravascular ultrasound-guided reverse overlapping stenting of a long calcified lesion using ultra-low contrast and metallic roadmaps: case report

**DOI:** 10.1093/ehjcr/ytad561

**Published:** 2023-12-18

**Authors:** Massoud A Leesar, Shao-Liang Chen

**Affiliations:** Division of Cardiology, University of Alabama, 510 20th street South, FOT-920 Birmingham, AL 35294, USA; Division of Cardiology, Nanjing First Hospital, Nanjing, China

**Keywords:** Calcified lesion, IVUS, Reverse overlapping stenting, Ultra-low contrast, Metallic roadmaps, Case report

## Abstract

**Background:**

Percutaneous coronary intervention (PCI) of a long calcified coronary lesion in patients with chronic kidney disease (CKD) is challenging and can lead to stent under-expansion and contrast-induced acute kidney injury (CI-AKI). We described the first case of intravascular ultrasound (IVUS)-guided reverse overlapping stenting of long calcified left anterior descending (LAD) coronary lesion using ultra-low contrast and the metallic roadmaps to prevent CI-AKI after PCI.

**Case summary:**

A 77-year-old man with a history of hypertension, type 2 diabetes, and CKD was admitted with angina class 4 and ruled in for non-ST-elevation myocardial infarction. His ejection fraction was 40%. He was referred for cardiac catheterization and PCI. Coronary angiography showed a long calcified stenosis of the LAD. IVUS catheter was advanced at least 10 mm distal to the lesion or stent edge. IVUS images were obtained with automated pullback (1 mm/s) using a commercially available IVUS system with a 60-MHz mechanical transducer (Boston Scientific, Natick, Massachusetts). IVUS showed calcified plaque fractures after balloon angioplasty and intracoronary lithotripsy. The first stent was deployed proximally using the guidewire in the diagonal branch as a metallic roadmap, and the second stent was deployed distally overlapping at the distal edge of the first stent as a roadmap with no contrast injection. Percutaneous coronary intervention was completed successfully using only 12 mL contrast. Glomerular filtration rate remained stable after PCI. Glomerular filtration rate and ejection fraction improved at 12-month follow-up.

**Discussion:**

We described the first case of the reverse overlapping stenting technique guided by IVUS with no contrast in a patient with CKD and a long calcified LAD lesion. Conventionally, in long lesions, the first stent is deployed distally and the second stent proximally, which requires contrast injection for stent deployment. We demonstrated that the above technique resulted in preventing CI-AKI and improving creatinine as well as ejection fraction at follow-up.

**Learning points**

- Patients with chronic kidney disease (CKD) and a long calcified coronary lesion are at increased risks of contrast-induced acute kidney injury, dialysis, morbidity, and mortality after percutaneous coronary intervention.
- We described the first case of the reverse overlapping stenting technique guided by intravascular ultrasound using ultra-low contrast and the metallic roadmaps in a patient with CKD and a long left anterior descending lesion.
- We demonstrated that the above technique resulted in preventing acute kidney injury and improving glomerular filtration rate as well as ejection fraction at follow-up.

## Introduction

Contrast-induced acute kidney injury (CI-AKI) after percutaneous coronary intervention (PCI) is associated with an increased risk of adverse events, including dialysis and death.^1,2^ Intravascular ultrasound (IVUS)-guided PCI can reduce contrast volume and the risk of CI-AKI.^3^ The technique of ultra-low contrast PCI (ULPCI) has been previously reported.^4^ However, the reverse overlapping stenting technique by IVUS guidance has not been investigated. In this case report, we described the first case of the reverse overlapping stenting technique guided by IVUS using ultra-low contrast and the metallic roadmaps in a patient with chronic kidney disease (CKD) and a long calcified left anterior descending (LAD) coronary artery lesion.

## Summary figure

**Table:** 

Timeline
On the day of admission, he was hydrated with normal saline. On the second day of admission, coronary angiography was performed using 10 mL contrast. The heart team approach was conducted and a decision was made to proceed with IVUS-guided PCI using ultra-low contrast and metallic roadmaps. He was continuously hydrated and PCI was performed on the third day of admission. Reverse overlapping stenting technique guided by IVUS was performed with no contrast and PCI was completed using 12 mL contrast (*Tables 1* and *2*). After 48 h of PCI, creatinine and glomerular filtration rate (GFR) remained unchanged and the patient was discharged. At 12-month follow-up, creatinine, GFR, and ejection fraction improved to 1.7%, 40%, and 55%, respectively.

### Case presentation

**Figure 1:**
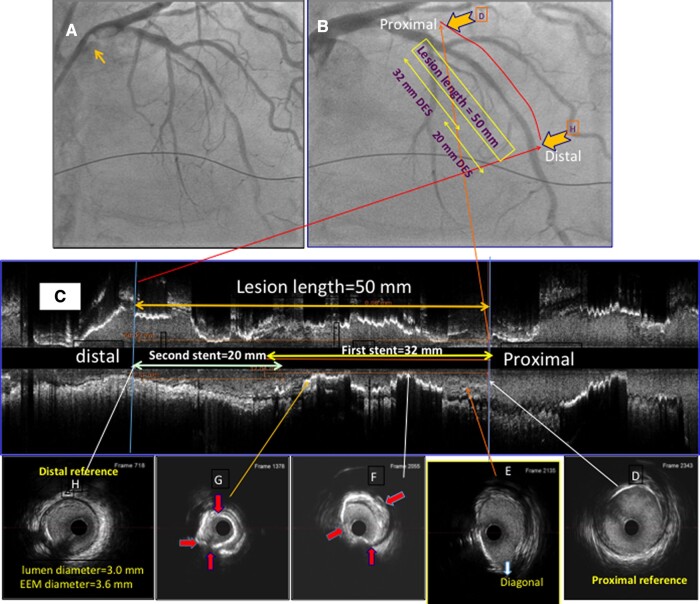
Coronary angiography and IVUS assessment: (*A*) Coronary angiography showing a critical calcified stenosis in the mid left anterior descending coronary artery; (*B*) the stenosis improved after non-compliant balloon and lithotripsy treatment; and (*C*) IVUS showing the lesion length was 50 mm from normal proximal (Frame D) to the distal reference (Frame H) with corresponding markers on coronary angiography (*B*). IVUS also shows fractures of calcified plaque (red arrows, Frames F and G) and distal reference dimensions (Frame H). Based on IVUS, two stents were required, a 3.0 × 32 mm stent to cover from Frame D (proximal reference) to pass Frame 4G (stenosis) and a 3.0 × 20 mm stent to overlap 2 mm with the proximal stent, as a metallic roadmap, and to extend to Frame H (distal reference).

A 77-year-old man with a history of hypertension, type 2 diabetes, and CKD (creatinine 2.4 mg/dL, GFR 29-mL/min/1.73 m^2^) was admitted with angina class 4 and ruled in for non-ST-elevation myocardial infarction. His ejection fraction was 40%. Coronary angiography showed a critical calcified stenosis of the LAD artery (*Figure 1A* and Supplementary material online, *Video S1*).

On the day of admission, he was hydrated with normal saline. On the second day of admission, coronary angiography was performed. He was continuously hydrated and ULPCI was performed on Day 3 of admission (Summary figure and *Table 1*). In order to minimize the contrast use, the still diagnostic image of the LAD in the right anterior oblique view was displayed on the monitor (*Figure 1A*). Ultra-low contrast PCI was performed using non-compliant (NC) and lithotripsy balloons with an improvement in stenosis (*Figure 1B*). Given the large profile of lithotripsy balloon, we first predilated the calcified lesion using high-pressure NC balloon inflation followed by shock wave lithotripsy. After lesion preparation with NC and lithotripsy balloons, IVUS was performed using a 60-MHz IVUS imaging system (Boston Scientific, Inc. Natick, Massachusetts) with automatic pullback at 1 mm/second to assure the calcified plaque was fractured prior to stent deployment. Given the above strategy, we did not face any resistance in advancing lithotripsy balloon, IVUS, or stent through the lesion.

**Table 1:** Step-by-step approach for hydration and the use of ultra-low contrast

**Figure 2:**
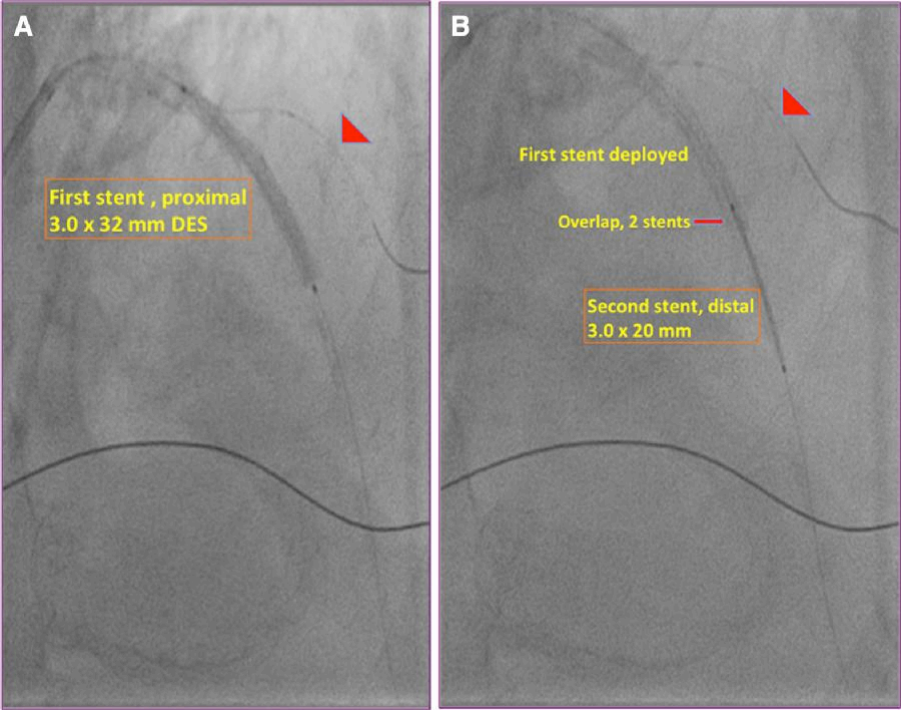
A novel technique of reverse overlapping stenting: (*A*) The first stent was deployed proximal to the guidewire (arrowhead) in the diagonal branch as a metallic roadmap, and (*B*) the second stent was deployed overlapping 2 mm distal to the first deployed stent as a metallic roadmap.

**Figure 3:**
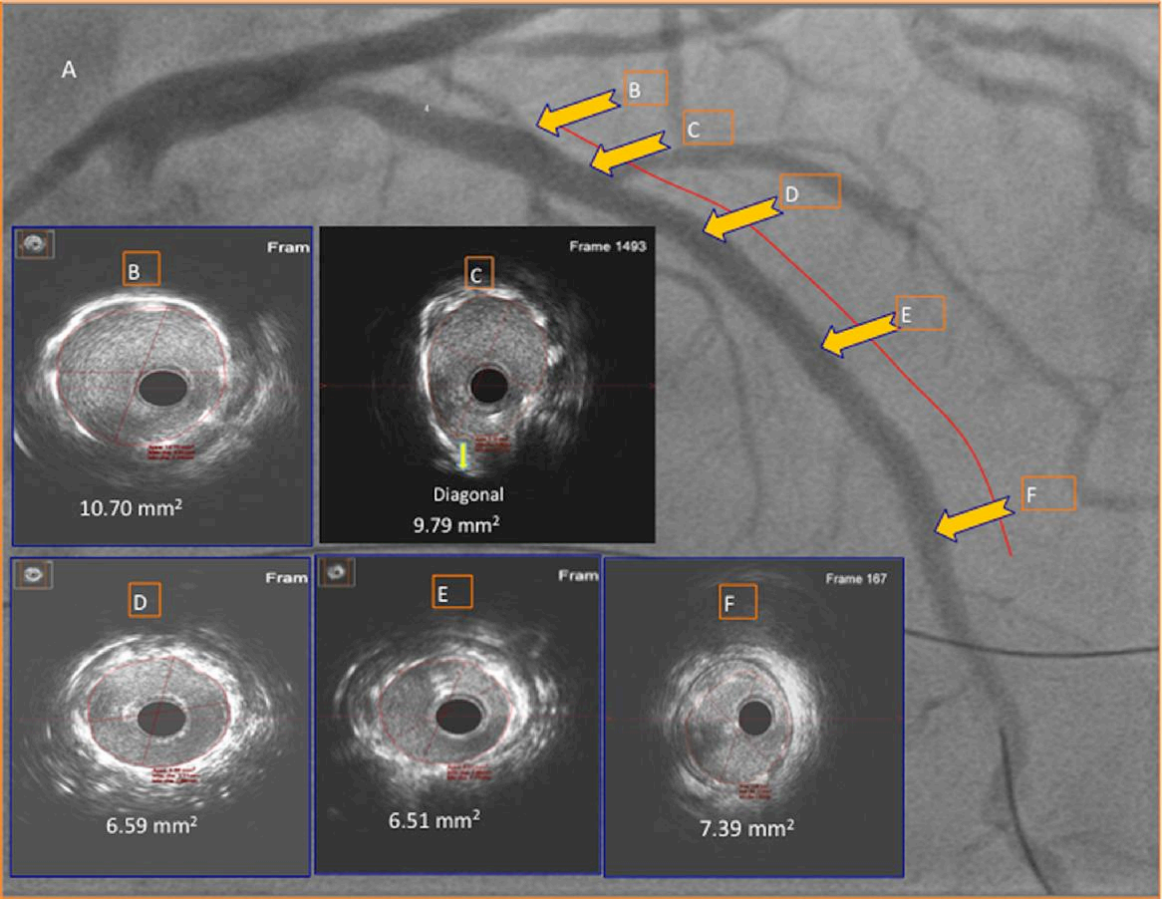
Final results: IVUS showing stents are optimally expanded (minimum stent area at the lesion site (Frame E = 6.51 mm^2^) with complete lesion coverage (Frames B to F with corresponding final angiogram).

Hydration with isotonic saline was started on the day of admission (1 mL/kg/h, 12 h before coronary angiography). On the second day of admission, coronary angiography was performed with limited views using only 10 mL contrast while hydration was continued. On the third day of admission ULC-PCI were performed and hydration was continued for 12 h to reduce the risk of acute kidney injury. The still diagnostic image in the right anterior oblique view was displayed on the monitor (*Figure 1A*). The guide engagement with no contrast (calcification landmark at the left main ostium; *Figure 1A*, arrow) and T wave inversion on ECG with saline injection (not shown). A low-osmolar contrast medium diluted at 50% was used for the essential parts of the procedure, including (i) to assess the result of angiography after balloon angioplasty and lithotripsy (*Figure 1B*) and (ii) to assess the final result (*Figure 3A*). The proximal and distal stents were deployed with no contrast use (*Figure 2A* and *B*).

IVUS of the LAD showed 360° calcific arch and plaque fractures after PCI (*Figure 1C*, Frames 1F and 1G, and Supplementary material online, *Video S2*). By IVUS, the normal proximal to distal reference length was 50 mm requiring two stents (*Figure 1C*). The technique of reverse stenting technique included deploying the first stent proximally in which the proximal marker of the first stent (3.0 × 32 mm) was positioned just proximal to the guidewire in the diagonal branch (DB), as a metallic roadmap (arrowhead), and deployed (*Figure 2A* and Supplementary material online, *Video S3*). The second stent (3.0 × 20 mm) was overlapped 2 mm at the distal edge of the proximal deployed stent, as a metallic roadmap, and deployed with no contrast injection (*Figure 2B* and Supplementary material online, *Video S4*).

Post-dilation and the proximal optimization technique were performed using 3.5 and 4.0 mm NC balloons, respectively. IVUS showed optimal stent expansion with complete lesion coverage (*Figure 3* and Supplementary material online, *Videos S5* and *S6*). Percutaneous coronary intervention was completed using 12 mL contrast. The total PCI time was 64 min. Glomerular filtration rate remained stable at 48 h. At 12-month follow-up, creatinine, GFR, and ejection fraction improved to 1.7%, 40%, and 55%, respectively.

## Discussion

To the best of our knowledge, this is the first case of the reverse overlapping stenting technique guided by IVUS using ultra-low contrast and the metallic roadmaps in a patient with CKD and a long calcified LAD lesion. We deployed the first stent proximally using the guidewire in the DB as a metallic roadmap, and the second stent was deployed distally overlapping with the distal edge of the first stent as a metal roadmap with no contrast injection. We used only 12 mL contrast to assess angiography after intracoronary lithotripsy and at the end of the procedure to assess the final result. Glomerular filtration rate remained stable (*Table 1*). At 12-month follow-up, the patient had no events and GFR and ejection fraction improved.

Contrast-induced acute kidney injury is very common in patients with advanced CKD. In the National Cardiovascular Data Registry Cath-PCI registry,^5^ patients with a GFR < 30 mL/min/1.73 m^2^ had a 26.6% incidence of CI-AKI, while 4.3% required in-hospital dialysis. Additionally, CI-AKI has been associated with higher risks of mortality, myocardial infarction, bleeding, dialysis, and stent thrombosis.^6,7^ The contrast volume is linearly associated with CI-AKI after PCI.^8,9^ In this respect, efforts should be made to develop and implement contrast-sparing protocols and to use IVUS, as shown in *Tables 1* and *2*, to prevent acute kidney injury. In the MOZART (Minimizing cOntrast utiliZation With IVUS Guidance in coRonary angioplasty) trial,^10^ the authors investigated the feasibility of utilizing IVUS on reducing the contrast volume. They showed that the use of IVUS, as compared with angiography, significantly reduced the total contrast volume.

Conventionally, in long lesions, the first stent is deployed distally in the coronary artery and the second stent proximally, which requires contrast to deploy stents. In order to eliminate the contrast use with stenting, the reverse stenting technique was used (*Table 2*). The technique is defined as deploying the first stent proximally. The length of the lesion from the segment proximal to the DB to the segment distal to the calcified plaque (*Figure 1C*, Frame G) was 32 mm by IVUS. Thus, a 3.0 × 32 mm stent was deployed. The distal stent (3.0 × 20 mm) was overlapped 2 mm at the distal edge of the proximal deployed stent and deployed with no contrast injection (*Figure 2B*). Given the distal stent would need to be passed through the deployed proximal stent, the proximal stent should be adequately expanded. In this respect, the IVUS assessment prior to the proximal stent deployment showed a good lesion preparation with plaque ruptures and lesion expansion, which resulted in proximal stent expansion. If the distal stent would not pass through the proximal stent, post-stent dilation or the use of a guide extension catheter would facilitate the passage of the distal stent.

**Table 2:** Step-by-step approach for reverse stenting guided by intravascular ultrasound

Proximal reference was determined by IVUS as a frame with no disease (Frame 1D) proximal to the DB (Frame 1E). Distal reference was determined by IVUS as the frame with no disease in the distal LAD (Frame 1H). The lesion length was determined as the distance between the proximal and distal reference (50 mm) (*Figure 1C*). Since the lesion length was 50 mm by IVUS, two stents were required to cover the entire lesion. The length of the first stent was determined by IVUS (32 mm) to span from the proximal reference (Frame 1D) to distal to the lesion (Frame 1G). Since the lesion was planned to be covered by the proximal stent, the distal stent (20 mm) should pass the proximal stent to the distal reference (Frame 1H) with no resistance. A guidewire was positioned in the DB to create a metallic roadmap (*Figure 2A* and *B*, arrowheads). The first stent (32 mm) was positioned proximal to the DB (arrowhead) and was deployed (*Figure 2A*). The second stent (22 mm) was passed through the proximal stent, overlapped 2 mm with the proximal stent, as a metallic road map, and deployed (*Figure 2B*). Final angiography and IVUS images show complete coverage of the lesion with good stent expansion (*Figure 3*). Given the IVUS assessment of the lesion length and metallic roadmaps, no contrast was used to deploy the stents.

Alternatively, co-registration could have been used to deploy the distal stent first flowed by the proximal stent deployment. However, it requires coronary angiography to co-register IVUS images with corresponding coronary angiography. In this respect, the contrast use with coronary angiography poses some limitations on the use of co-registration in patients with severe CKD.

The strength and educational value of this approach relate to hydration with normal saline prior to the procedure, staging the procedure, lesion preparation with NC and lithotripsy balloons, IVUS assessment for plaque fracture, lesion length, stent sizing, and final results. In addition, the use of the reverse stenting technique for stent deployment with no contrast prevented acute kidney injury and improved outcome (*Table 2*). The limitation of this approach is that the above protocol will need to be investigated in a study.

## Conclusions

We described the first case of the reverse overlapping stenting technique guided by IVUS using ultra-low contrast and the metallic roadmaps in a patient with CKD and a long calcified LAD lesion. We demonstrated that the technique resulted in preventing acute kidney injury and improving GFR as well as ejection fraction at follow-up.

## Supplementary Material

ytad561_Supplementary_Data

## Data Availability

The data underlying this article are available in the article and in its online Supplementary material.

## Lead author biography

Dr Massoud A. Leesar is a professor of Medicine at the University of Alabama, Birmingham.

## Supplementary material

**Consent:** The authors confirm that written consent for submission and publication of this case including images and associated text has been obtained from the patient in compliance with COPE guidelines.

**Funding:** None declared.
