# Supplementary material for: The first reported case of intravascular ultrasound-guided reverse overlapping stenting of a long calcified lesion using ultra-low contrast and metallic roadmaps: case report
Source: Eur Heart J Case Rep. 2023 Dec 18;8(1):ytad561. doi: 10.1093/ehjcr/ytad561 (PMC10762894; doi:10.1093/ehjcr/ytad561)
Supplement: ytad561_Supplementary_Data [file ytad561_supplementary_data.zip › Supplementary Material_Reverse stenting.docx]

**Supplementary Material**

**The First Reported Case of IVUS-Guided Reverse Overlapping Stenting of a Long Calcified Lesion Using Ultra-Low Contrast and Metallic Roadmaps**

Massoud A Leesar^1^, MD; Shao-Liang Chen^2^ MD

1. Division of Cardiology, University of Alabama, Birmingham

2. Division of cardiology, Nanjing First Hospital, Nanjing, China

**Supplementary Videos**

1. Supplemental Video 1. Coronary angiography of the LAD. The angiogram demonstrates a critical stenosis in the mid LAD with severe calcification.

2. Supplemental Video 2. IVUS after balloon angioplasty and lithotripsy. Automatic IVUS pullback recordings from the distal LAD to diagonal (DA) show a segment with a 360**^ο^** calcific arch and plaque fractures after PCI.

3. Supplemental Video 3. The technique of reverse overlapping stenting (1). The first stent (3.0 x 32 mm) was positioned in a segment proximal to the diagonal branch (DA) using the gudiewire in DA (arrowhead) as a fiducial marker and deployed with no contrast injection.

4. Supplemental Video 4. The technique of reverse overlapping stenting (2). The second stent (3.0 x 20 mm) was deployed overlapping 2 mm at the distal edge of first stent as a fiducial marker with no contrast injection.

5. Supplemental Video 5. Final coronary angiography after stenting. The angiogram shows optimal results were achieved by angiography.

6. Supplemental Video 6. Final IVUS. IVUS demonstrates optimal stent expansion in the calcified segment.
